# Synthesis and Characterization of New Thieno[3,2-*b*]thiophene Derivatives

**DOI:** 10.3390/molecules171012163

**Published:** 2012-10-16

**Authors:** Moawia O. Ahmed, Wojciech Pisula, Subodh G. Mhaisalkar

**Affiliations:** 1 Department of Chemistry, Faculty of Science, Taibah University, P.O. Box 30003, Medina Munawarah 41477, Saudi Arabia; 2 Max Planck Institute for Polymer Research, Ackermannweg 10, D-55128 Mainz, Germany; 3 School of Materials Science and Engineering, Nanyang Technological University, 50 Nanyang Avenue, 639798 Singapore

**Keywords:** Thieno[3,2-*b*]thiophene, 2,5-dibromothieno[3,2-*b*]thiophene, 4-dodecyl-phenylboronic acid, 4-(trifluoromethyl)phenylboronic acid

## Abstract

Three derivatives of thieno[3,2-*b*]thiophene end-capped with phenyl units have been synthesized and characterized by MALDI TOF mass spectroscopy, elemental analysis, UV-vis absorption spectroscopy and thermogravimetric analysis (TGA). All compounds were prepared using Pd-catalyzed Stille or Suzuki coupling reactions. Optical measurements and thermal analysis revealed that these compounds are promising candidates for p-type organic semiconductor applications.

## 1. Introduction

In recent years, there has been growing interest in conjugated organic materials due to their interesting electronic properties [1,2,3,4,5,6,7,8]. Many studies on small molecules have been focused on pentacene, which has been used as an effective p-type semiconductor for organic field-effect transistors (OFETs) [9,10,11]. However, pentacene suffers from the disadvantage of being easily oxidized and it also presents extreme insolubility in common organic solvents. On the other hand, devices based on oligothiophenes have poor air stability and show rapid device-performance degradation due to their low band gap and high-energy of the highest occupied molecular orbital (HOMO) level [12,13,14]. Therefore, high stability as well as high charge carrier mobility of the OFET is desirable. It was an aim of the present work to design and synthesize some new organic materials with good air and thermal stability as semiconductors, using a straightforward synthetic route. We report the synthesis and characterization of a series of small compounds based on thieno[3,2-*b*]thiophene for thin-film trasistor (TFT) applications.

## 2. Results and Discussion 

**Scheme 1:**
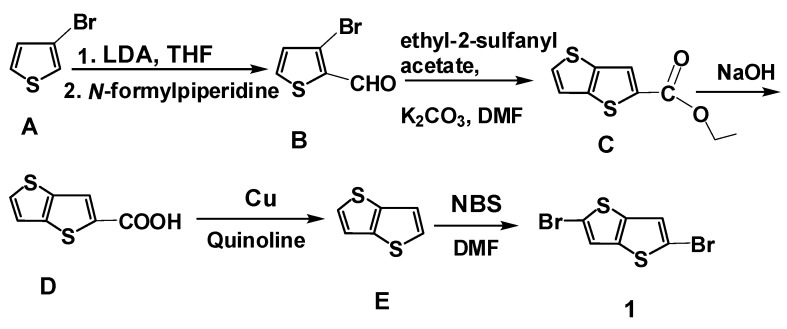
Preparation of **D**, **E** and **1**.

Thieno[3,2-*b*]thiophene-2-carboxylic acid (**D**) and thieno[3,2-*b*]thiophene (**E**) were prepared according to the method of Fuller and co-workers from commercially available 3-bromothiophene (**A**) [15]. 2,5-Dibromothieno[3,2-*b*]thiophene (**1**) was prepared according to the procedures reported by Xinnan and co-workers (Scheme 1) [16]. Compounds **D**, **E** and **1** were characterized by ^1^H- and ^13^C-NMR and GC-mass spectroscopic techniques (Figures S1 and S2).

**Scheme 2:**
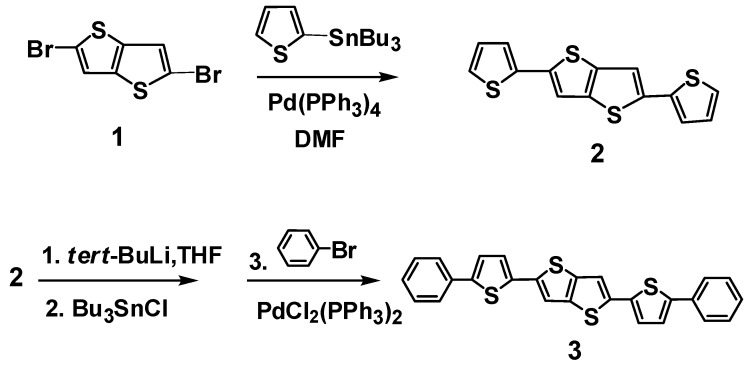
Synthesis of** 3** by Stille coupling reaction.

**Scheme 3:**
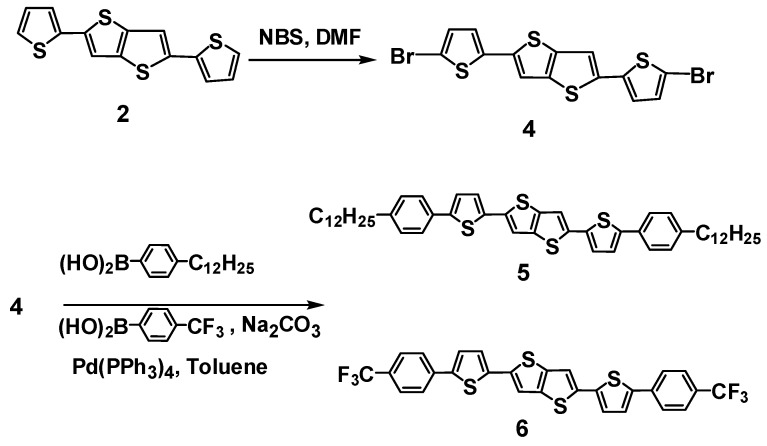
Synthesis of **5** and **6** by Suzuki coupling reaction.

The syntheses of compounds **3**, **5** and **6** were achieved as outlined in Scheme 2 and Scheme 3. Compound **3** was synthesized by utilizing a one-pot Stille coupling between bromobenzene and the freshly prepared tri-*n*-butylstannyl derivative of the known compound 2,5-bis(5-(tributylstannyl)thiophen-2-yl)thieno[3,2-*b*]thiophene** (2**) in the presence of catalytic PdCl_2_(PPh_3_)_2_ in refluxing THF (Scheme 2) [17].

**Figure 1:**
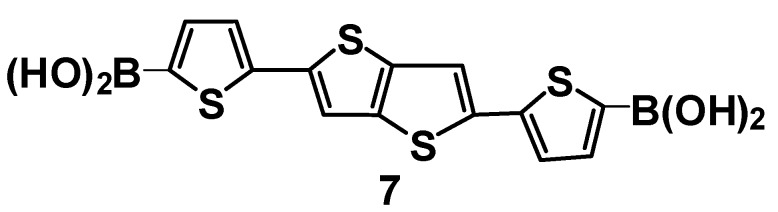
5,5′-(thieno[3,2-b]thiophene-2,5-diyl)bis(thiophene-5,2-diyl)diboronic acid (**7**).

Compounds **5** and **6** were synthesized by the organometallic Suzuki coupling of 2,5-bis(5-bromo-thiophen-2-yl)thieno[3,2-*b*]thiophene (**4**) and the appropriate arylboronic acid [4-dodecyl-phenylboronic acid for **5** or 4-(trifluoromethyl)phenylboronic acid for **6**] using Pd(PPh_3_)_4_ in refluxing THF (Scheme 3). The Stille coupling reaction was used instead of the Suzuki one to synthesize compound **3** because our attempts to synthesize pure 5,5′-(thieno[3,2-b]thiophene-2,5-diyl)bis(thiophene-5,2-diyl)diboronic acid (**7**, Figure 1) from compound **2** were unsuccessful. This was probably due to the relatively poor stability of the thiophene boronic acid derivatives, which decreases as the number of thienyl units increases [18]. Thanks to the one-pot Stille coupling reaction, which showed good efficiency and with a proper mixing of bromobenzene and freshly prepared tri-*n*-butylstannyl derivative of compound **2**, we were able to obtain the desired compound **3** in good yield and with high purity after vacuum sublimation.

All compounds are solid yellow in color and have very limited solubility in common organic solvents. Therefore, it is hard to characterize them by NMR techniques. This is in sharp contrast to their recently reported analogues with two alkyl chains introduced at the position 3- or 4-position of the thiophene ring which possess good solubility in common organic solvents and are easily characterized by ^1^H and ^13^C-NMR techniques [19,20]. Nevertheless, the solubility of compounds **3**, **5** and** 6** in THF was just high enough to investigate their optical properties. Compounds **3**, **5** and **6** were purified by vacuum sublimation and their masses were determined by MALDI TOF spectroscopy. Prominent base peaks for the molecular ions were observed for all compounds in the mass spectra (Figure S3). 

**Figure 2:**
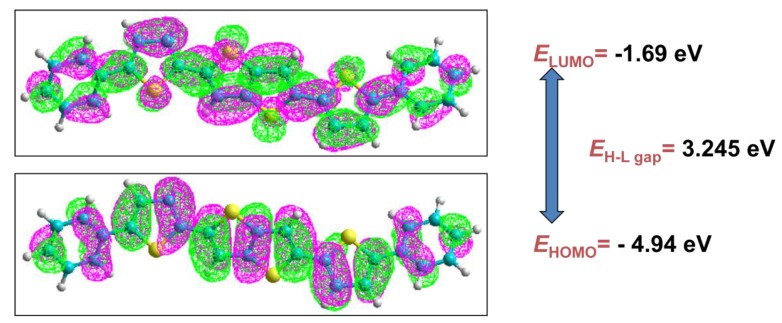
HOMO and LUMO Compound **3**.

To gain a better understanding of the various factors influencing the stability of the synthesized molecules, the highest occupied molecular orbital (HOMO) and the lowest unoccupied molecular orbital (LUMO) of **3** (Figure 2) were determined by complete geometry optimization using the gradient-corrected B3LYP density functional with the polarized 6-31G * basis set (B3LYP/6-31G *) [21]. The calculated HOMO and LUMO energies are listed along with the associated band gap in Figure 2 and Figure S4. The calculated HOMO level of **3** is lower than those of most oligothiophenes and pentacene, indicating a higher oxidative stability [11,14]. 

**Figure 3:**
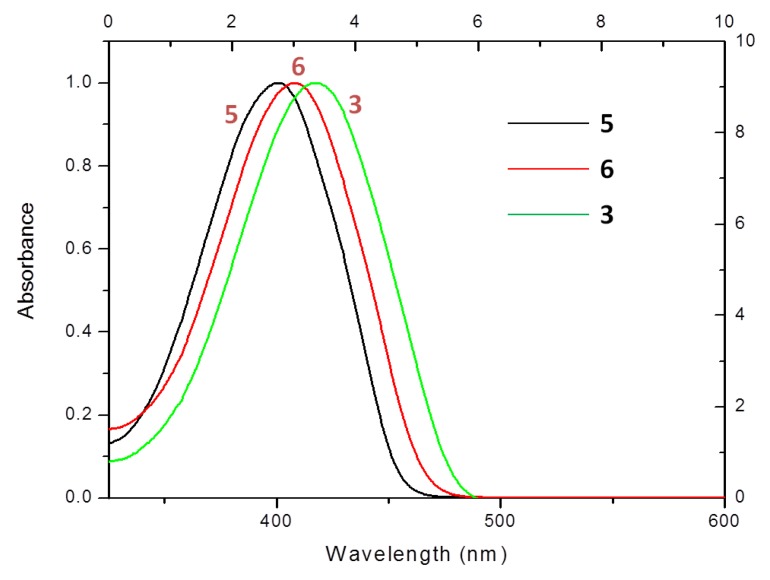
UV-Vis spectra of **3**, **5** and **6**.

The optical properties of **3**, **5** and **6** were investigated by UV-vis absorption spectroscopy. The absorption spectra of the three compounds are depicted in Figure 3. Compounds **3**, **5** and **6** exhibited absorption maxima in THF solution at 415, 401, and 408 nm, respectively. 

The absorption maximum of unsubstituted compound **3** is red shifted compared to that of α,ω-dodecyl and trifluoromethyl substituted derivatives **5** and **6**, respectively. The estimated HOMO-LUMO gaps obtained from the end absorptions are 2.50 eV for **3**, 2.65 eV for **6** and 2.80 eV for **5**. The lower gap for **3 **is probably due to the lesser steric hindrance between the two phenyl rings and thiophene units, which leads to more effective π-conjugation and stronger interactions between the molecules of **3**. On the other hand, the presence of substituents in **5 **and **6** leads to the more steric hindrance which leads in turn to less effective π-conjugation and weaker interactions between molecules. The absorption maximium of compound **6** (408 nm) in THF solution is red-shifted with respect to that of *β,β *'-dodecyl substituted derivative **8** (389 nm) recently reported by our group indicating the presence of strong intermolecular interactions between **6** molecules. The estimated HOMO-LUMO gaps obtained from the end absorptions for **6** (2.65 eV) is lower compared to that of **8** (2.71 ev). The variation of the energy gaps may indicate a different influence of the dodecyl substituents on packing and device performance of these materials. 

**Figure 4:**
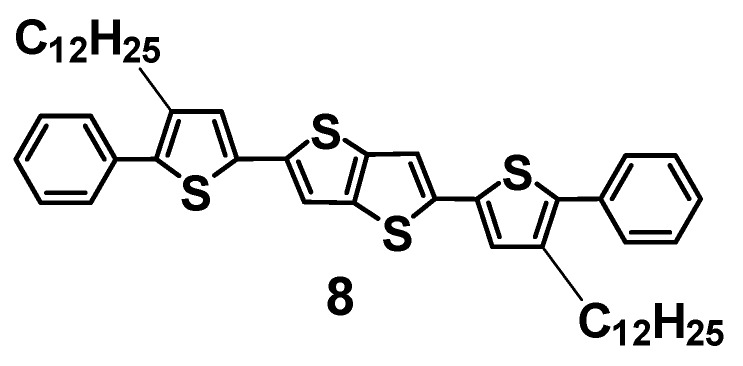
Compound **8**.

Moreover, The HOMO level of unsubstitued derivative **3** (−4.94 eV) and **8** (−5.25 eV) match well with the workfunction of metallic gold (−5.1 eV) and this may enhance the hole charge injection between the electrode and the semiconductor and may greatly improve the device performance. The presence of long alkyl chains in compound **8** (Figure 4) improves its solubility as well as its molecular ordering, thus positively influencing its semiconducting properties. 

**Figure 5:**
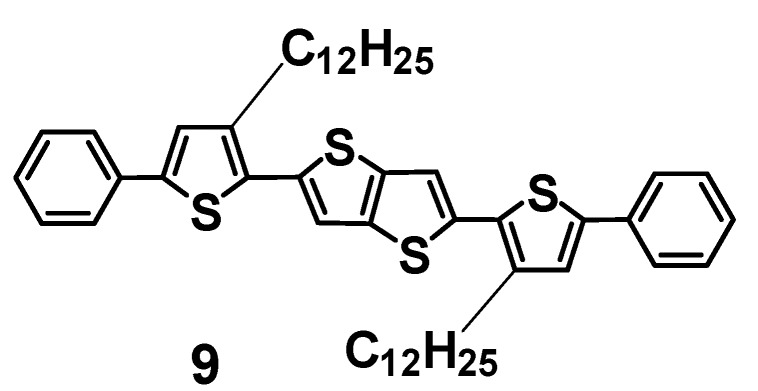
Compound **9**.

In our previous investigations we showed that OTFT devices based on compound **8** have good stability and with a carrier mobilities up to 3.11 × 10^−2^ cm^2^ V^−1^ s^−1^ and on/off ratios up to 10^4^ for a top-contact OTFT made by spin coating. In contrast, compound **9** (Figure 5) showed extremely poor FET performance, which may be due to a lower degree of molecular ordering caused by substituent positions as evidenced by DSC data and two-dimensional wide-angle X-ray scattering results [19]. 

**Figure 6:**
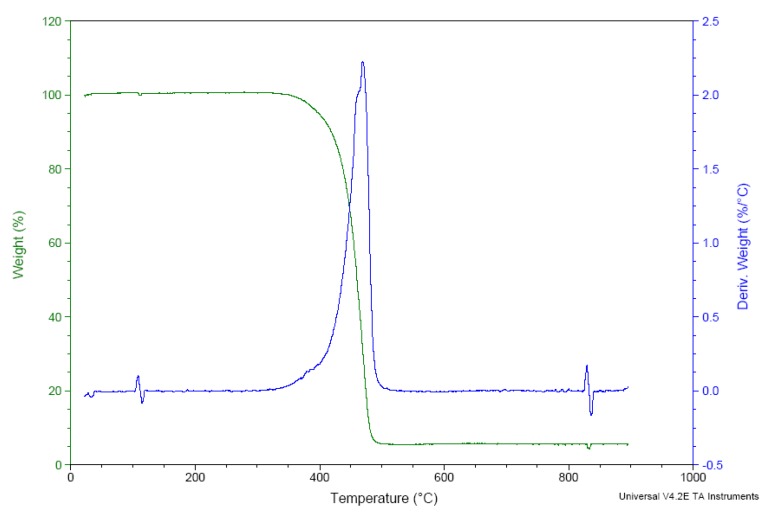
Thermal gravimetric analysis of **5**.

Compounds **3**, **5** and **6** possess limited solubility in common organic solvents, which makes them promising candidates for organic electronic devices prepared by vapor evaporation. Thermogravimetric analysis (TGA) reveals as all compounds being thermally stable, with decomposition temperatures above 350 °C under nitrogen atmosphere. Figure 6 shows the TGA for **5**.

## 3. Experimental 

### 3.1. General 

Reactions and manipulations were performed using standard Schlenk techniques under an atmosphere of nitrogen or argon, unless otherwise stated. Solvents were purified, dried, and distilled under an argon or nitrogen atmosphere prior to use. All new compounds were characterized by MALDI TOF mass spectrometry and elemental analysis. ^1^H and ^13^C-NMR spectra were recorded on a Bruker DPX 400 MHz spectrometer with chemical shifts referenced to CDCl_3_. Thermogravimetric analyses (TGA) were conducted on a Du Pont Thermal Analyst 2100 system with a TGA 2950 thermogravimetric analyzer under a heating rate of 20 °C/min and a nitrogen flow rate of 70 mL/min. 

### 3.2. Materials 

Lithium diisopropylamide (LDA), *N*-formylpiperidine, ethyl-2-sulfanyl acetate, bromobenzene, tributyl(thiophen-2-yl)stannane, thieno[3,2-*b*]thiophene, *tert*-butyllithium, tri-*n*-butyltin chloride, PdCl_2_(PPh_3_)_2_, tetrakis(triphenylphosphine)palladium, *N*-bromosuccinimide (NBS), 4-(trifluoro-methyl)phenylboronic acid and 4-dodecylphenylboronic acid, were all purchased from Aldrich (Singapore). All reagents purchased commercially were used without further purification except for tetrahydrofuran (THF), which was dried over sodium–benzophenone ketyl.

### 3.3. Synthesis 

Thieno[3,2-b]thiophene-2-carboxylic acid (**D**), thieno[3,2-*b*]thiophene (**E**), 2,5-dibromothieno[3,2-*b*]thiophene (**1**), 2,5-di(thiophen-2-yl)thieno[3,2-*b*]thiophene (**2**) and 2,5-bis(5-bromothiophen-2-yl)thieno[3,2-*b*]thiophene (**4**) were prepared according to the procedures reported in the literature [15,16,17].

*2,5-Di(thiophen-2-yl)thieno[3,2-b]thiophene* (**2**). A mixture of 2,5-dibromothieno[3,2-b]thiophene (**1**) (2.80 g, 9.33 mmol), tributyl(thiophen-2-yl)stannane (8.01 g, 21.46 mmol), and tetrakis(triphenyl-phosphine)palladium(0) (0.33 g, 0.467 mmol) in dry DMF (50 mL) was heated under nitrogen at 90 °C overnight. After the reaction was cooled to room temperature, water (100 mL) was added to the solution and the yellow precipitate was collected by filtration, washed with hexanes and ether, and then purified by vacuum sublimation to afford 1.80 g of the tile compound as a yellow solid (63%). MALDI TOF-MS (dithranol) *m/z*: 303.904 (100, M+), calcd for C_14_H_8_S_4_ = 309.950. Calcd for C_14_H_8_S_4_: C 55.23, H 2.65; S 42.13; Anal. found: C 55.87, H 2.54; S 42.07.

*2,5-Di(thiophen-2-yl)thieno[3,2-b]thiophene* (**3**). Compound **2** (2.00 g, 6.58 mmol) was dissolved in dry THF (50 mL) and cooled in an acetone/dry ice bath for 20 minutes. *n*-Butyllithium (1.60 M solution in hexanes, 8.22 mL, 13.16 mmol) was then added dropwise and the mixture was allowed to stir 1 h. At this point the mixture was removed from the ice bath, allowed to warm to room temperature, and then returned to the ice bath. Tributyltin chloride (3.57 mL, 13.16 mmol) was then added dropwise and the mixture was allowed to warm to room temperature and stirred for 1 h, the resulting 2,5-bis(5-tributylstannylthien-2-yl)thieno[3,2-*b*]thiophene solution was added by cannula under nitrogen to a solution PdCl_2_(PPh_3_)_2_ (0.40 g, 0.57 mmol) and bromobenzene (1.77 g, 11.33 mmol) in THF (20 mL). The mixture was heated to reflux under argon for 20 h. The solution was then allowed to cool, quenched with water, and the orange solid was isolated by filtration and successively washed with water, 1 N HCl, and acetone (25 mL each). The material was purified by vacuum sublimation (*p* < 10^−5^ Torr), giving a bright yellow solid 1.20 g (40%). MALDI TOF-MS (dithranol) *m/z* (relative intensity): 455.955 (100, M^+^), 456.971 (30%), 457.937 (20%); calcd. for C_26_H_16_S_4_ = *m/z*: 456.01 (100.0%), 457.02 (28.3%), 458.01 (1 8.1%). Anal. calcd for C_26_H_16_S_4_: C 68.38, H 3.53, S 28.09; found: C 68.57, H 3.39, S 27.84.

*2,5-Bis(5-bromothiophen-2-yl)thieno[3,2-b]thiophene* (**4**). N-Bromosuccinimide (1.75 g, 9.87 mmol) was added in portions over 20 min to a solution of **2** (1.50 g, 4.93 mmol) in DMF (20 mL) at 0 °C. After stirring the solution for 3 h at ambient temperature, water (50 mL) was added, and the aqueous layer was extracted with ethyl acetate (50 mL). The organic layer was dried (anhydrous Na_2_SO_4_) and the solvent removed *in vacuo*. The residue was purified by chromatography on silica gel eluting with hexanes to give **4** as a yellow solid (1.90 g, 83% yield). MALDI TOF-MS (dithranol) *m/z*: 461.797 (100%) 463.797 (62.00%) and 459.797 (47.00%) (M); calcd. for C_14_H_6_Br_2_S_4_ = 461.770 (100.0%), 463.770 (61.50%), 459.770 (46.90%). Anal. Calcd for C_14_H_6_Br_2_S_4_: C, 36.38; H, 1.31; S, 27.75. Found: C, 36.69; H, 1.18; S, 27.84.

### 3.4. General Method for the Preparation of ***5*** and ***6***

To a deoxygenated solution of 4-dodecylphenylboronic acid (1.35 g, 4.50 mmol) or 4-(trifluoromethyl)phenylboronic acid (0.86 g, 4.50 mmol) and Na_2_CO_3_ (20.00 mL; 2.00 M), a deoxygenated solution** 4** (0.80 g, 1.73 mmol) in toluene (60.00 mL) and tetrakis(triphenylphosphine)-palladium(0) (0.10 g, 0.087 mmol) was added. The reaction mixture was refluxed for 24 h under argon atmosphere. The cooled solution was poured into a flask containing water (150 mL) mixed with 1.00 M HCl (30 mL) and toluene (200 mL). The aqueous and organic phases were separated; the orange solid was isolated by filtration and washed with water. The product was washed with toluene and dried under vacuum. The product was further purified by sublimation to yield **5** (0.95 g, 69%) as a yellow crystals and **6** (0.76 g, 74%) as orange crystals. 

(**5**) MALDI TOF-MS (dithranol) *m/z*: 792.389(M); calcd. for C_50_H_64_S_4_ = 792.390. Anal. Calcd for C_50_H_64_S_4_: C, 75.70; H, 8.13; S, 16.17. Found: C, 75.96; H, 8. 05; S, 16.10.

(**6**) MALDI TOF-MS (dithranol) *m/z*: 591.791(M); calcd. for C_28_H_14_F_6_S_4_ = 591.990. Anal. Calcd for C_28_H_14_F_6_S_4_: C, 56.74; H, 2.38; S, 21.64. Found: 56.55; H, 2.52; S, 21.49. 

## 4. Conclusions

In summary, we have demonstrated the synthesis and characterization of a new family of thieno[3,2-*b*]thiophene derivatives. Their photophysical and thermal analysis were investigated. All compounds exhibited good thermal stability and appear therefore to be promising candidates for p-type organic semiconductor applications. Further studies on the solid state packing of compounds **3**, **5** and **6** using two-dimensional wide-angle X-ray scattering (2D-WAXS) and device performance in OFETs are under way. 

## Supplementary Materials

Supplementary materials can be accessed at: http://www.mdpi.com/1420-3049/17/10/12163/s1.
