# Supplementary material for: Synthesis and Characterization of New Thieno[3,2-b]thiophene Derivatives
Source: Molecules. 2012 Oct 16;17(10):12163–71. doi: 10.3390/molecules171012163 (PMC6269117; doi:10.3390/molecules171012163)

Supplementary Materials

Figure S1. (**a**) ^{1}H-NMR of thieno[3,2-*b*]thiophene-2-carboxylic acid (**D**); (**b**) ^{13}C-NMR of thieno[3,2-*b*]thiophene-2-carboxylic acid (**D**); (**c**) ^{1}H-NMR of thieno[3,2-*b*]thiophene (**E**).

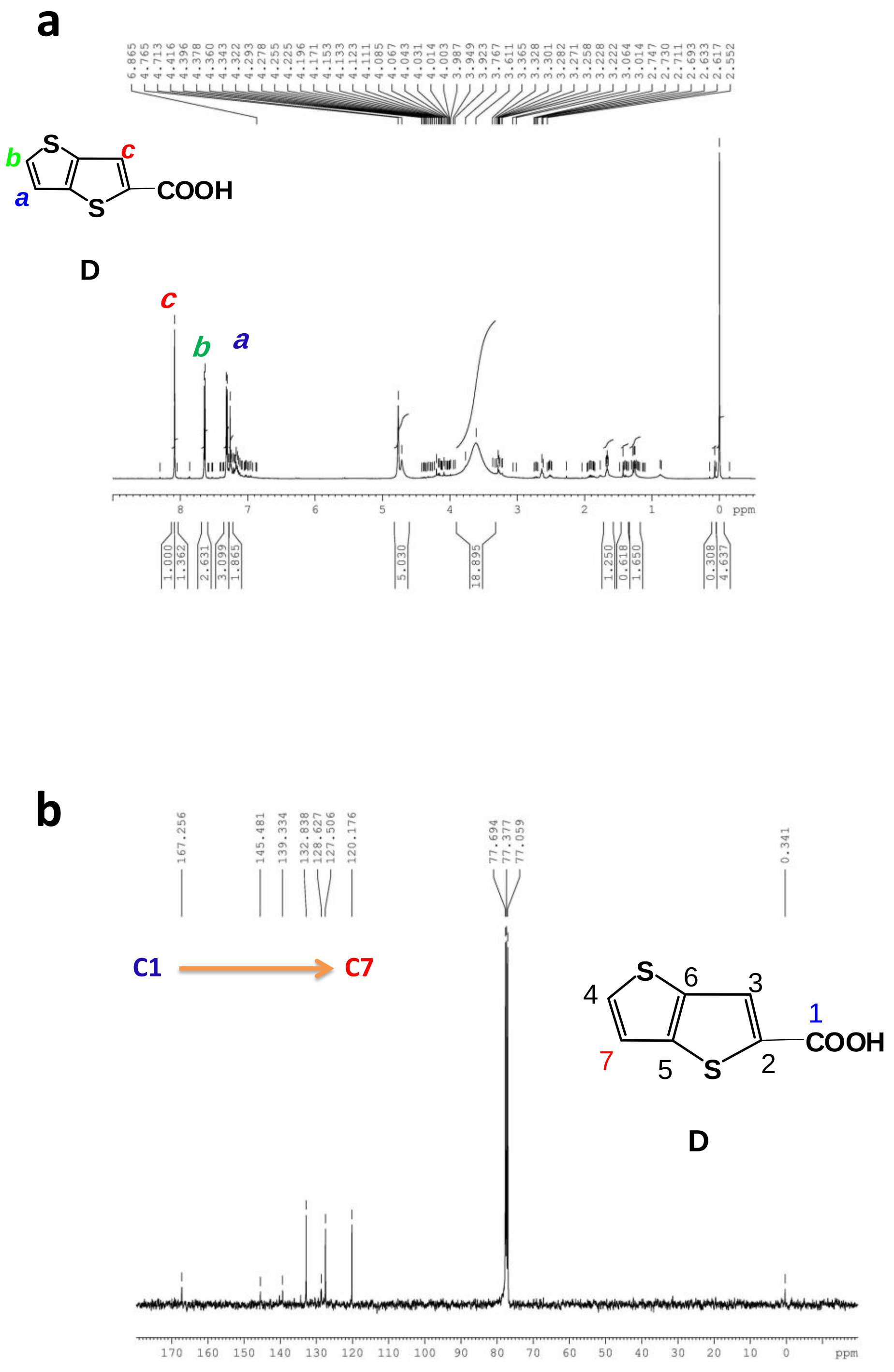

Figure S1. *Cont.*

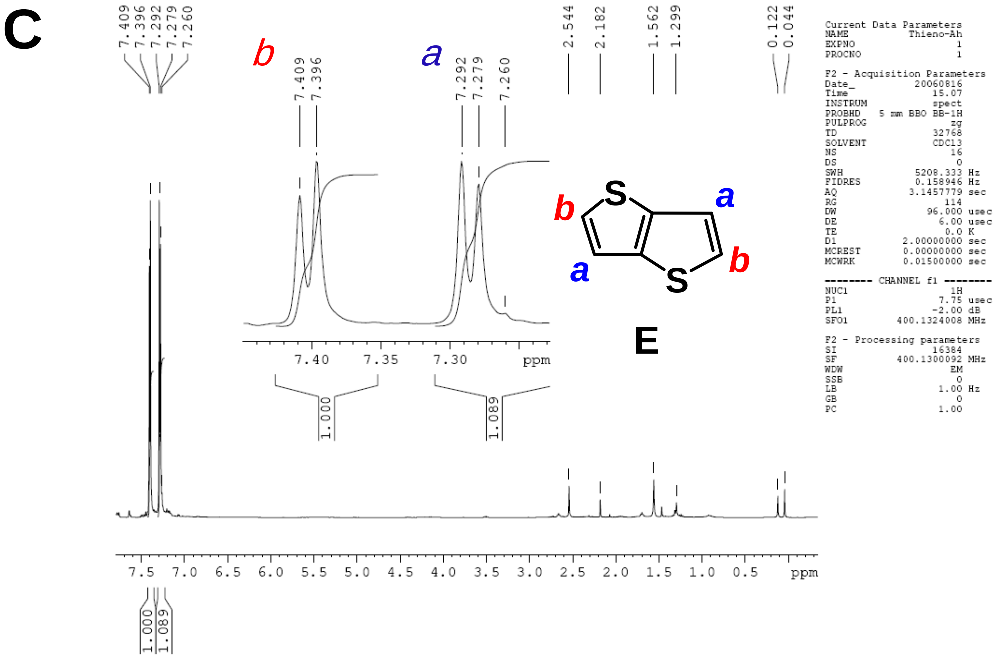

Figure S2. (**a**) GC-mass spectra of 2,5-dibromothieno[3,2-*b*]thiophene (**1**); (**b**) mass spectrum of 2,5-dibromothieno[3,2-*b*]thiophene (**1**).

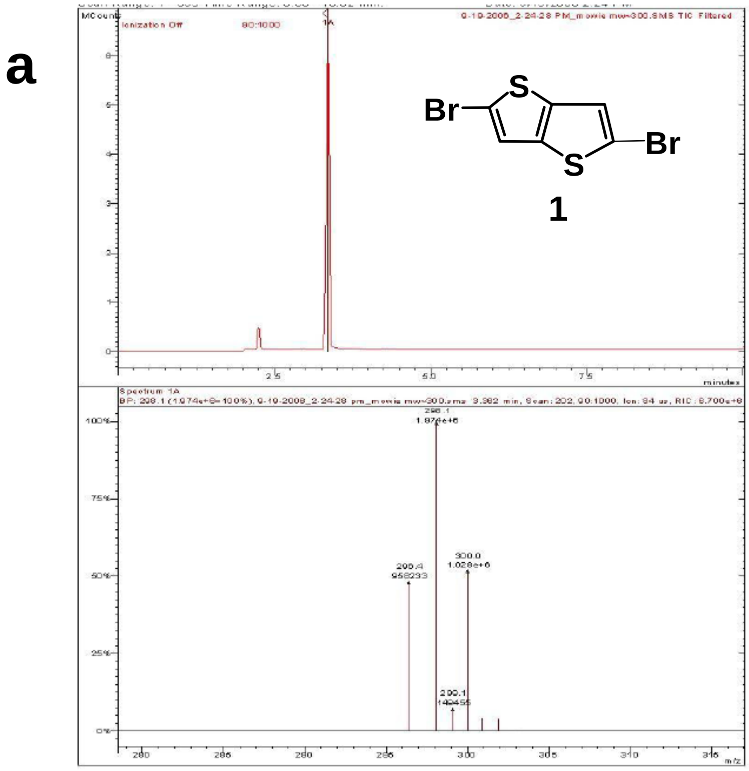

Figure S2. *Cont.*

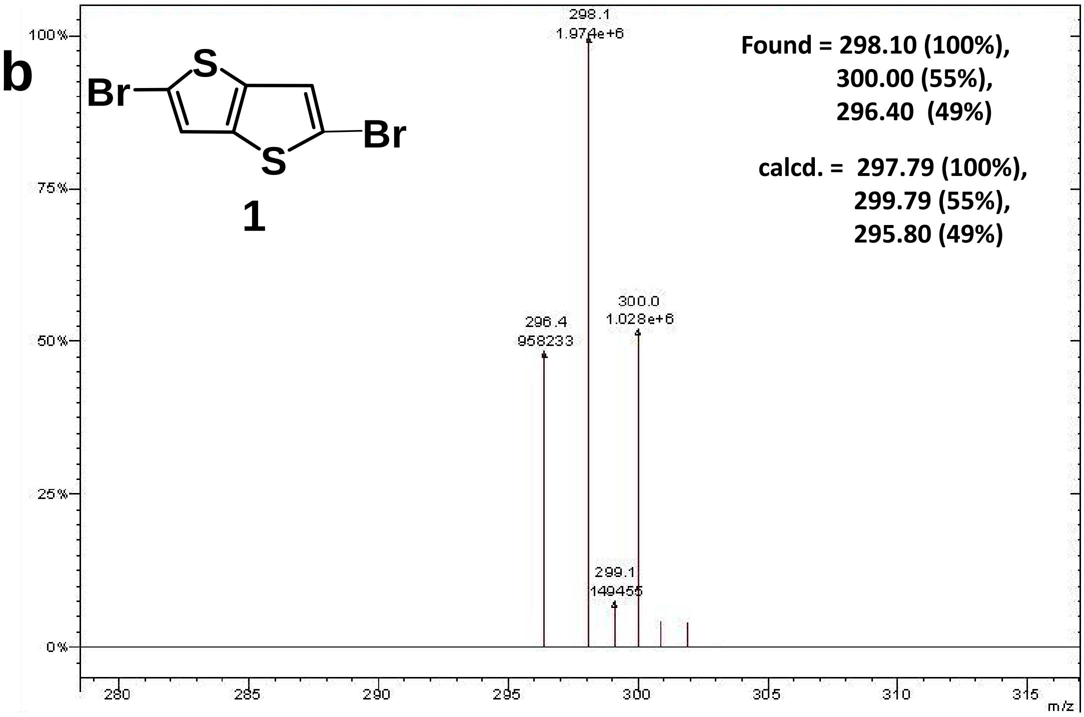

Figure S3. MALDI TOF mass spectra of **2**–**6**.

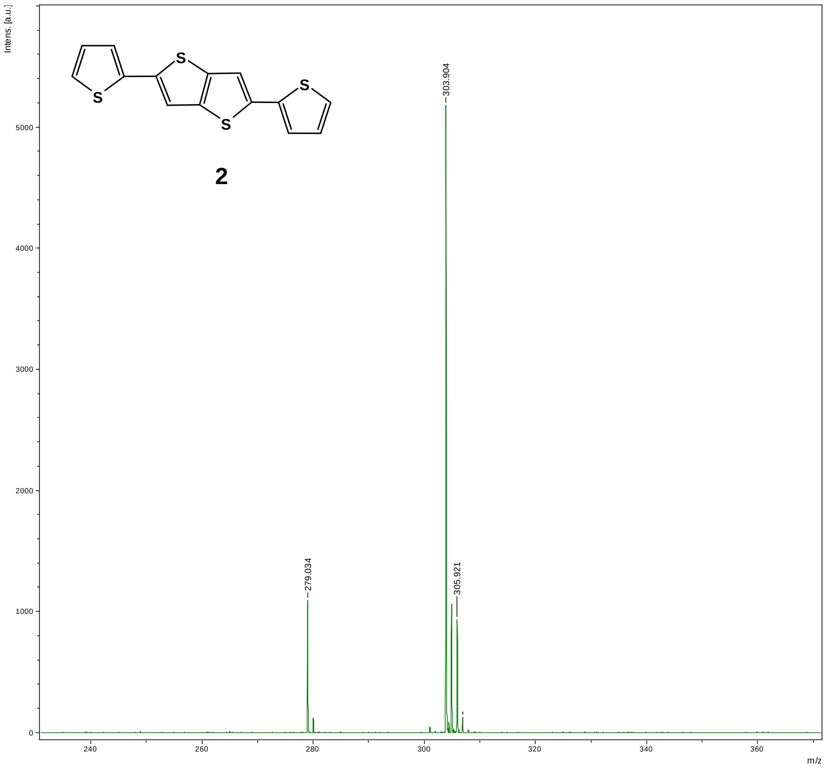

Figure S3. *Cont.*

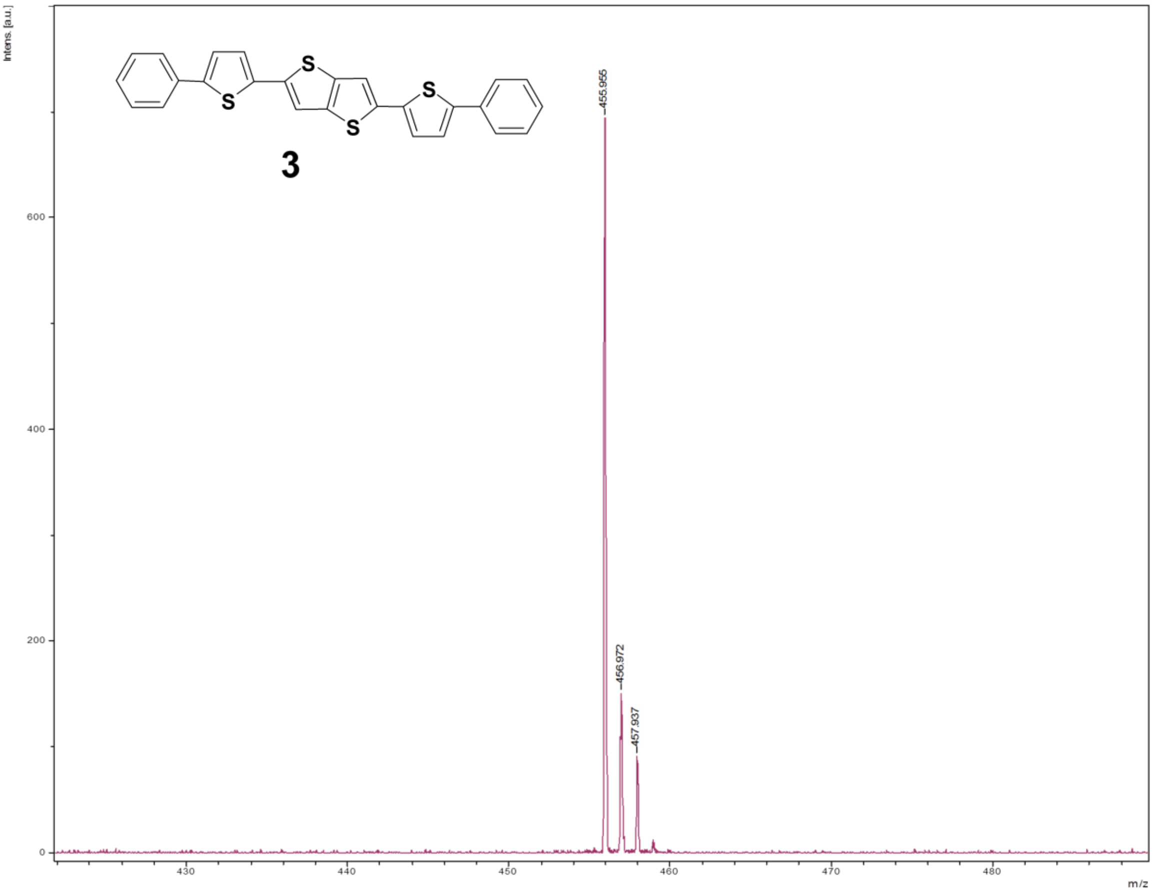

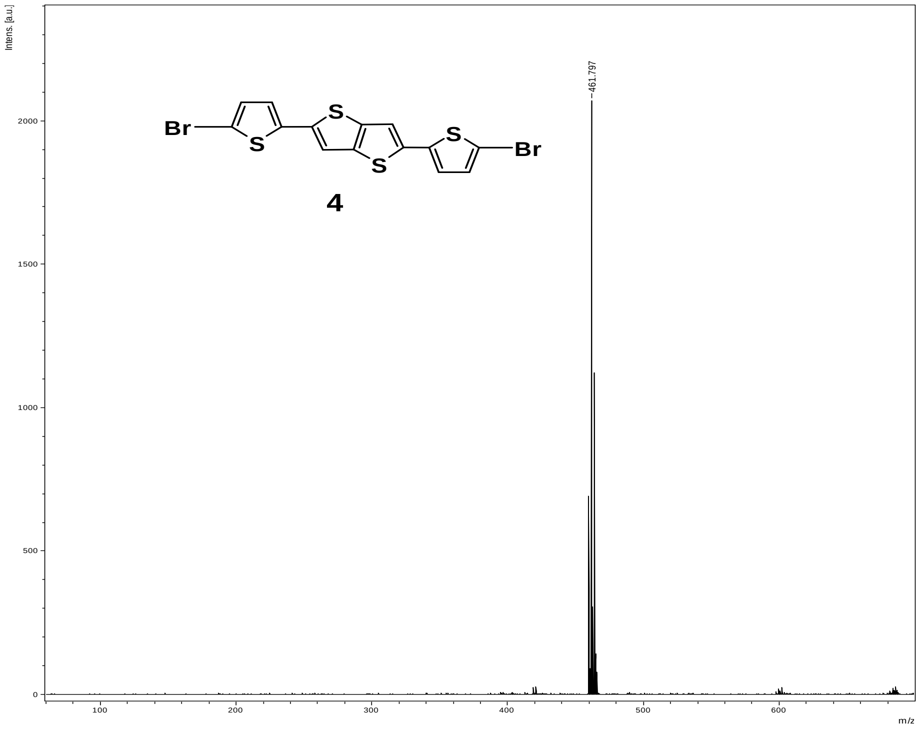

Figure S3. *Cont.*

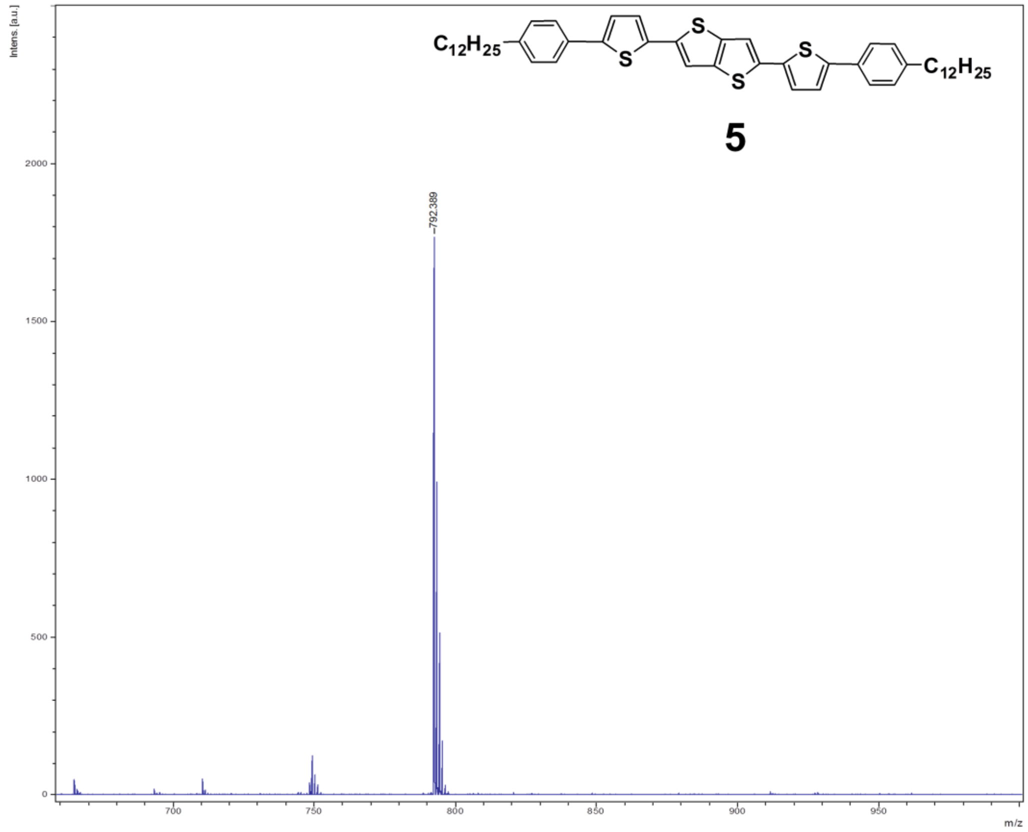

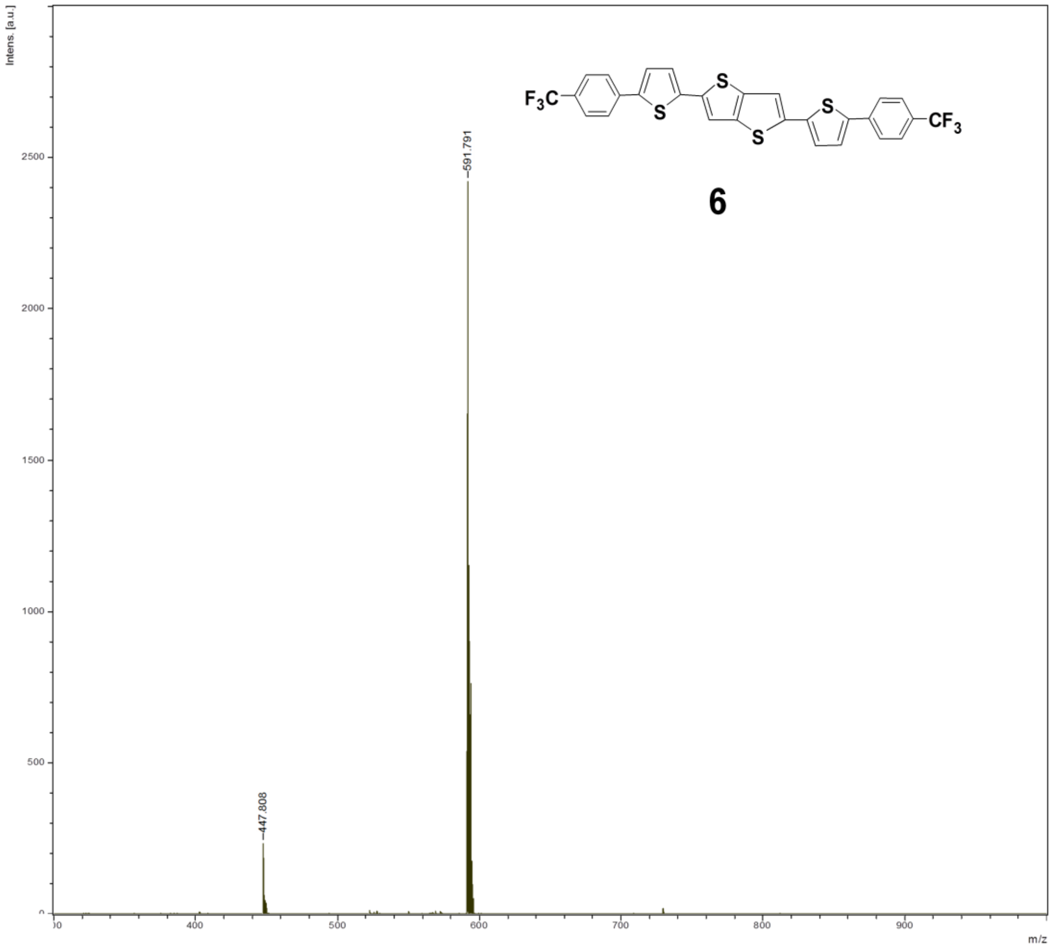

Supplement: Supplementary file 1 [file molecules-17-12163-s001.pdf]
